# Integrative biochar and melatonin application mitigates lead toxicity in rice by modulating antioxidant activities and iron plaque formation and downregulating the expression of metal uptake genes

**DOI:** 10.3389/fpls.2025.1609825

**Published:** 2025-07-03

**Authors:** Tahir Abbas Khan, Qitao Su, Huang Guoqin, Zhixuan Du, Mehmood Ali Noor, Tahani A. Y. Asseri, Muhammad Umair Hassan

**Affiliations:** ^1^ Research Center on Ecological Sciences, Jiangxi Agricultural University, Nanchang, China; ^2^ School of Life Sciences, Key Laboratory of Jiangxi Province for Biological Invasion and Biosecurity, Jinggangshan University, Ji’an, China; ^3^ Key Laboratory of Crop Physiology, Ecology and Genetic Breeding, Ministry of Education, Jiangxi Agricultural University, Nanchang, China; ^4^ King Khalid University, College of Science, Department of Biology, Abha, Saudi Arabia

**Keywords:** antioxidants, biochar, melatonin, metal availability, translocation, yield

## Abstract

Lead (Pb) is a common toxic metal that causes severe health and environmental problems. However, the defensive role and underlying mechanism of combined biochar (BC) and melatonin (MT) against Pb stress are still unclear. Therefore, to fill this gap, this study investigated the impacts of BC and MT on rice growing in Pb-polluted soil. This study included different treatments: control, Pb stress (300 mg kg^−1^), Pb stress (300 mg kg^−1^) + BC (2%), Pb stress (300 mg kg^−1^) + MT (30 µM), and Pb stress (300 mg kg^−1^) + BC (2%) + MT (30 µM). Pb reduced rice growth and yield by hindering photosynthetic pigments, relative water contents (RWCs), osmolyte synthesis, nutrient uptake, increase in oxidative markers, and Pb accumulation. Biochar and MT increased rice productivity by increasing chlorophyll synthesis, osmolytes, and nutrient uptake and decreasing Pb accumulation. The co-application of BC and MT decreased Pb accumulation in the roots (30.40%) and shoots (72.79%), the translocation factor (30.01%), the biological accumulation coefficient (20.17%), and the soil Pb concentration (59.02%). The co-application of BC and MT enhanced proline (39.65%), soluble protein (47.09%), ascorbate peroxidase (APX; 26.47%), catalase (CAT; 65.51%), peroxidase (POD; 89.56%), and superoxide dismutase (SOD; 65.53%) activities, which ensured better productivity. Additionally, the BC+MT application increased the expression of antioxidant defense genes (*OsAPX*, *OsCAT*, *OsPOX*, and *OsSOD*) and decreased the expression of metal transporter genes (*OsHMA9* and *OsNRAMP5*), which protected the rice plants from damage caused by Pb toxicity. These results suggested that BC+MT could be a promising strategy to mitigate Pb toxicity and maintain sustainable and safer food production.

## Graphical Abstract

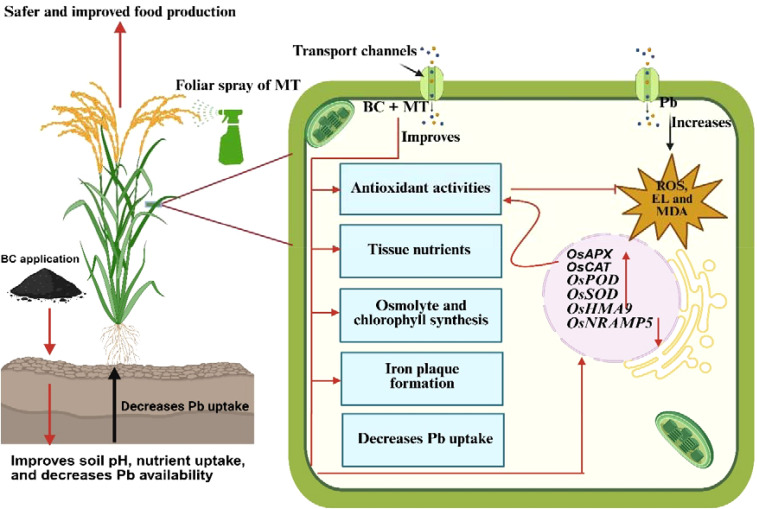

## Introduction

Heavy metal (HM) pollution is a global issue due to its hazardous impacts on plants, living organisms, and ecosystems (Kaur et al., 2019). The concentrations of heavy metals are continuously increasing, which is a major threat to crop productivity (Saddique et al., 2018; Jalil et al., 2023). Heavy metals enter the soil via wastewater, smelting, industrial discharge, eroding, and crumbling, which negatively impact soil properties and microbial activities (Ali et al., 2019). Lead (Pb) is an extremely toxic HM that enters the environment through diverse sources of sewage sludge, smelting, mining, and Pb-based products including paints, pulp, and gasoline (Raj and Maiti, 2020). It is a non-biodegradable metal and is considered the second most toxic element, following arsenic (Zhang et al., 2024), which negatively affects plants and humans and poses serious health and environmental concerns (Wen et al., 2024).

Pb is a toxic metal, and its natural concentration in soil ranges from 10 to 40 mg kg^−1^; however, recent industrial development and human activities have significantly increased its concentration in soils (Ghouri et al., 2024). It is a toxic metal for plants, and it inhibits plant growth when it is present at concentrations greater than 30 mg kg^−1^ (Usman et al., 2020). Plants quickly absorb Pb and accumulate it in their organelles, which increases reactive oxygen species (ROS) production, which causes oxidation of lipids, membranes, and proteins (Xu et al., 2021; Nawaz et al., 2023), resulting in a marked decrease in plant growth (Li et al., 2020). It also impedes chlorophyll synthesis, nutrient and water uptake, and root growth (Zanganeh et al., 2021). Elevated levels of Pb also inhibit enzyme activity and alter membrane permeability, water balance, and mineral nutrition (Fan et al., 2020; Maneechakr and Mongkollertlop, 2020). Moreover, it also damages photosystem-II (PS-II), blocks the energy transfer pathway between amino acids, and reduces the absorption of visible light (Aslam et al., 2021).

Rice is a staple food for many nations; however, it easily accumulates toxic metals including Pb owing to long-term flooding conditions (Lü et al., 2021). The European Commission and World Health Organization set a permissible limit of 200 μg kg^−1^ in rice grains to protect human health (Åkesson et al., 2015; European Commission Regulation (EU), 2015). Pb causes serious health issues (Shaheen et al., 2022; Yang et al., 2023a); thus, it is essential to develop measures to restrict its mobility and availability to safeguard better human health (Yang et al., 2023b). Biochar is an excellent strategy used globally for mitigating the impacts of toxic metals (Gu et al., 2023). It possesses excellent functional groups, porous structure, and resistance to decomposition, which makes it an important amendment for mitigating the impacts of toxic metals (Chen et al., 2022a). Biochar application substantially decreases Pb bioavailability, which limits Pb uptake and decreases its accretion in plants (Chen et al., 2023). It also decreases Pb accumulation in diverse organelles at the subcellular level and mitigates Pb toxicity by increasing antioxidant defense and osmolyte synthesis (Zhou et al., 2016). Additionally, biochar (BC) also decreases ROS production, protects cellular membranes and photosynthetic apparatus, and maintains better nutrient and water uptake, leading to improved growth under Pb toxicity (Chen et al., 2023).

Biochar is used with different amendments to enhance its efficiency to mitigate the toxic impacts of HM. For instance, it is used with organic manure, hormones, and microbes to increase its efficiency against toxic metals (Haider et al., 2022). Melatonin (MT) is a non-toxic molecule with tremendous potential to improve plant performance in stressful conditions (Hoque et al., 2021). It improves seed germination, stand establishment, and nutrient and water uptake, thereby mitigating the toxic impacts of stress conditions (Samanta et al., 2021). Melatonin improves plant physiological functioning, antioxidant activities, and osmolyte synthesis, thus increasing the plant’s resistance to stress (Kaya et al., 2022). It also improves gene expression and positively influences soil microbial activities, which favors plant growth in stress conditions (Yu et al., 2022). Although many studies have determined the impacts of BC on rice in Pb-polluted soils, there is a knowledge gap concerning the effects of the BC+MP application to mitigate the hazardous impacts of Pb. Therefore, we hypothesized that the combination of BC and MT could effectively immobilize Pb and decrease its toxicity to rice. The aims of this study were to i) assess the influence of BC and MT on rice growth and physiological and biochemical functions, ii) assess the effects of BC and MT on Pb availability and accumulation in soil–rice systems, and iii) determine the impacts of BC and MT applications on antioxidants and metal uptake gene expression and iron plaque formation.

## Materials and methods

### Study site

**Figure 1:**
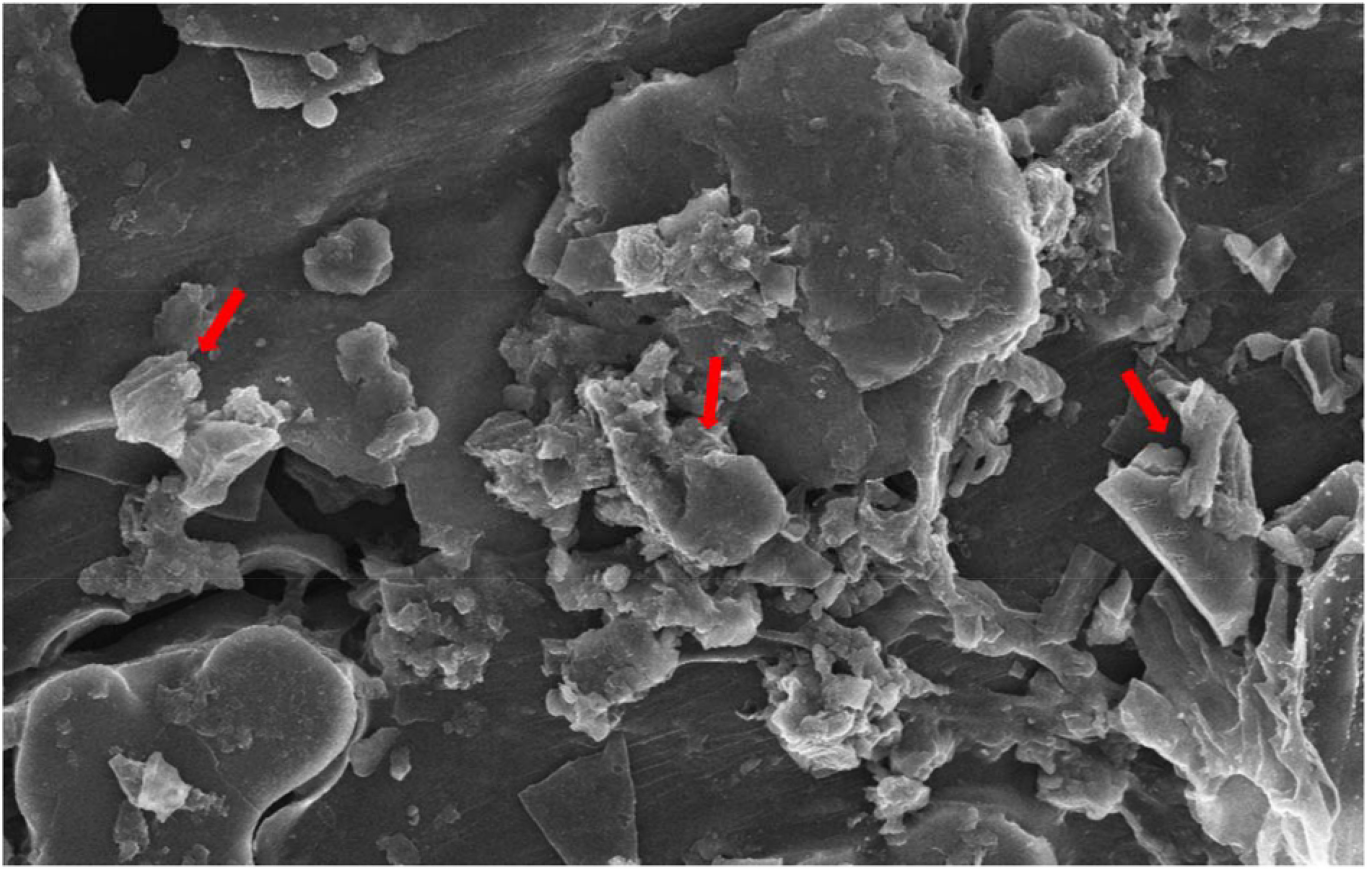
Scanning electron microscopy analysis of biochar used in the study.

The study was performed in an open greenhouse with an artificial rain shelter at Jiangxi Agricultural University Nanchang, China (28°46′ N, 115°36′ E) in 2024. This site has a sub-tropical humid climate with an annual temperature of 17°C and an annual precipitation of 1,741 mm. The soil for the experiment was taken from the experiment field, which has a silty loam texture with an acid pH of 5.45, total nitrogen (TN; 1.62 g kg^−1^), and available phosphorus (AP) and potassium (AK) contents of 31.22 and 114.33 mg kg^−1^, respectively. Pots were filled with 10 kg of dry soil, and Pb was applied according to the specific treatment plan. The source of Pb was PbCl_2_, and it was mixed with and stabilized for 1 month. Thereafter, soil from every pot was taken, BC was added, and soil pots were filled again; water was applied to five 25-day-old seedlings sown in every pot. Rice straw was collected, and BC was prepared via pyrolysis of straws for 8 hours at 600°C. The biochar contained a significant amount of carbon (611 g kg^−1^), a cation exchange capacity of 10.13 cmol kg^−1^, and a nitrogen concentration of 3.98 g kg^−1^ and had an alkaline pH of 9.72. Furthermore, BC was also subjected to scanning electron microscopy analysis. Biochar used in the current study showed porous structures with rough surfaces and aromatic structures with a high degree of amorphous mass disorder (**Figure 1**).

### Treatments and crop husbandry

The study included the following treatments: control, Pb stress (300 mg kg^−1^), Pb stress (300 mg kg^−1^) + BC (2%), Pb stress (300 mg kg^−1^) + MT (30 µM), and Pb stress (300 mg kg^−1^) + BC (2%) + MT (30 µM). The study was conducted in a completely randomized design (CRD) with three replications. The pots were regularly monitored, and a water depth of 3–4 cm was maintained throughout the growing period. The foliar spraying of MT was conducted after 20 days of transplanting. Foliar spraying was performed using a hand sprayer until the plants became fully wet. The plants were fully acclimatized after 20 days of transplantation, and they developed a sufficient area for the absorption of MT. Therefore, MT was applied after the 20th day of transplanting.

### Physiological traits

The plant samples were collected at the flag leaf stage (45 days after transplanting) for the analysis of physiological and biochemical traits. Leaves were collected and weighed to determine fresh weight (FW), and then they were water-soaked for 24 hours and weighed again to determine turgid weight (TW). Then, these leaves were oven-dried (24°C) and weighed to determine the dry weight (DW), and finally, relative water content (RWC) was determined with the following equation as suggested by Chattha et al. (2022): (FW − DW)/(TW − DW) × 100. For assessing electrolyte leakage (EL), rice leaves were incubated at 25°C for 30 minutes, and electrical conductivity (EC_1_) was measured. Then, these leaves were again incubated for 24 hours at 90°C, EC_2_ was measured, and finally, EL was determined following the procedures of Chattha et al. (2022) and using the following formula: EL = EL_1_/EC_2_ × 100. To determine photosynthetic pigments, 0.5-g leaf samples were ground in 80% acetone solution and centrifuged (10,000 rpm) to obtain the supernatant. Then, the concentrations of chlorophyll-*a*, chlorophyll-*b*, and carotenoid were estimated by reading absorbance at 663, 645, and 480 nm, respectively (Lichtenthaler, 1987) with the following formulas:


Chlorophyll a=(12.7(OD663)2.69(OD645))V/1,000

Chlorophyll b=(22.9(OD645)4.68(OD663))V/1,000 W

Carotenoid=[(OD480)+0.114(OD663)0.638(OD645)]/2,500

Here, W indicates the weight of the sample, while V is the volume of the supernatant.

### Oxidative stress markers and osmolytes

Fresh leaf samples (0.5 g) were ground in trichloroacetic acid (TCA; 5%) solution and centrifuged (10,000 rpm) for 15 minutes to collect the supernatant. Subsequently, 1 mL of the supernatant was combined with 1 mL of potassium iodide buffer (PIB) and 100 µL of phosphate buffer, and the absorbance was measured at 390 nm (Velikova et al., 2000). The malondialdehyde (MDA) concentration was determined according to the protocols of Rao and Sresty (2000). Fresh leaf samples (0.5 g) were ground using TCA (5%) solution and centrifuged (15,000 rpm) for 15 minutes, and MDA was estimated by measuring absorbance (532 nm). For total soluble protein (TSP), fresh leaves (0.5 g) were ground in 5 mL of potassium phosphate buffer (PPB; 50 mM) and homogenized (12,000 rpm) for 15 minutes. Then, 3 mL of Bradford reagent and 100 µL of plant extract were combined and placed at room temperature conditions, and absorbance was measured (590 nm; Bradford, 1976). For free amino acids (FAA), the supernatant was obtained by grinding 0.5-g fresh leaves in PPB. The 1 mL of collected supernatant and 1 mL of both ninhydrin and pyridine were mixed and incubated (90°C), and absorbance (570 nm) was measured by following the procedures of Hamilton and VanSlyke (1943).

### Antioxidant activity

The fresh leaves (0.5 g) of rice were ground in chilled PPB (50 mM) and centrifuged (12,000 rpm) at 4°C, after which the supernatant was collected. To measure ascorbate peroxidase (APX), 100 µL of enzyme extract was taken; 700 µL of buffer (50 mM), H_2_O_2_ (6.1 mM), and ascorbic acid (0.5 M) was added; and later, absorbance was read at 290 nm (Asada and Takahashi, 1987). For catalase (CAT), 0.1 mL of supernatant was added with 2.5 mL of buffer and 0.1 mL of H_2_O_2_, and absorbance was measured at 240 nm (Chance and Maehly, 1955). For peroxidase (POD), 100 µL enzyme extract was mixed with 700 µL PPB and 100 µL H_2_O_2_ (300 mM), and the absorbance was measured at 470 nm to determine POD activity (Chance and Maehly, 1955). For superoxide dismutase (SOD), enzyme extract (50 μL) was mixed with 25 mL of PPB, H_2_O_2_ (400 µL), Triton (100 µL), and 50 µL of riboflavin and nitro blue tetrazolium (NBT), and the absorbance was measured at 560 nM (Zhang, 1992).

### Determination of nutrients and Pb in plant tissues and soil properties

The plant samples were ground and digested with HNO_3_:HClO_4_ (2:1) on a hot plate (Jones and Case, 1990), and the Pb concentration was measured via atomic absorption spectrometry. The concentration of N was measured using the Kjeldahl procedure, whereas P was assessed using a spectrophotometer. Moreover, calcium (Ca), potassium (K), and magnesium (Mg) concentrations in roots and shoots were determined using a flame photometer. A soil and water paste (1:5) was made, and a pH meter was used to measure the soil pH. Soil organic carbon (SOC) was measured via the sulfuric acid–potassium dichromate external heating technique. The concentration of available phosphorous (AP) was measured using the sodium bicarbonate extraction procedure following the Olsen method (Olsen et al., 1982). The soil available potassium (AK) was determined via the ammonium acetate extraction technique (Helmke and Sparks, 1996). The soil TN concentration was assessed using the Kjeldahl method as suggested by Bao (2000). To determine the soil Pb concentration, the soil samples were digested with HClO_4_ and HNO_3_ at 160°C. Then, the samples were allowed to cool and filtered, and the Pb concentration was assessed via atomic absorption spectrophotometry. The biological accumulation coefficient (BAC) and translocation factors (TFs) were determined via the procedures of Malik et al. (2010). Different fractions of Pb were assessed via the sequential extraction technique based on the European Community Bureau of Reference (BCR) (Xiong et al., 2018). This method measures the following fractions of Pb: exchangeable, reducible, oxidizable, and residual.

### Growth and yield traits

Five plants were harvested at the maturity stage (90 days after transplanting) to measure growth and yield traits. The height of five plants from each pot was measured using a measuring tape, and then the average was taken. The same plants were carefully uprooted, the roots and shoots were separated, and the roots were washed carefully to remove the soil. The root length was measured in centimeters using a measuring tape. The roots were weighed on an electric balance to determine their fresh weight. Then, roots were oven-dried (65°C) until constant weight to determine their dry weight (g). The plants from the weighed pot were harvested and weighed to determine biomass yield, and later, grains were separated and weighed to determine the grain yield. Both grain and biomass yields were measured in grams. Furthermore, 100 grains were taken to determine the grain weight (g), and the harvest index was determined as the ratio of grain to biomass yield.

### Determination of iron plaque formation and expression analysis of antioxidants and metal uptake genes

Fresh root samples were collected, and iron plaque formation on the roots was determined via the ascorbic citrate acetic (ACA) extraction technique. For this purpose, an ACA mixture containing ascorbic acid (3 g), 5 mL of sodium acetate (10%), and 40 mL of sodium citrate (0.3 M) was prepared. Thereafter, 1-g root sample was collected, placed in the prepared mixture, and shaken (280 rpm) for 3 hours at 25°C. The mixture was subsequently filtered into 100-mL flasks, and the roots were carefully washed three times. The eluent obtained after washing the roots was transferred to the same 100-mL flasks, and the volume was increased to 100 mL by adding the water. The concentrations of Fe and Pb were determined via atomic absorption spectrophotometry.

The plant samples (100 mg) were collected, and RNA was extracted using an RNA extraction kit (MiniBEST; Takara, China) following the protocols of the manufacturer. Thereafter, both the quality and concentration of RNA were determined using a NanoDrop spectrophotometer. A transcription kit (HiScript^®^ III RT SuperMix) was used to convert the RNA into cDNA. To perform real-time PCR, 11 μg of extracted RNA was reverse-transcribed into cDNA using a Dispelling RT, SuperMix kit. Later, qRT–PCR was performed using a SuperReal PreMix Plus (SYBR Green) kit (Tiangen, Takara, China; FP205–2). Additionally, OsActin was used as a reference gene, and relative gene expression was calculated using the methods of Livak and Schmittgen (2001). The copper/zinc SOD was targeted from the SOD gene family, and in the case of POD, the POD from the peroxidase family was targeted. Moreover, CAT was targeted from the catalase–peroxidase family, and APX was targeted from the ascorbate peroxidase family. The details of the primers used in the study are given in **Supplementary Table S1**.

### Statistical analysis

The data were analyzed by one-way analysis of variance to determine the impact of different treatments. Moreover, differences among means were analyzed using Tukey’s honestly significant difference (HSD) test, Sigma-plot 10 was used for figure preparation, and principal component analysis (PCA) and correlation matrix were plotted in RStudio.

## Results

### Chlorophyll and relative water contents

Pb toxicity decreased chlorophyll, carotenoid, and RWC (**Table 1**). However, BC and MT applications offset this decrease and caused a marked improvement in the chlorophyll contents and RWC (**Table 1**). The co-application of BC and MT enhanced Chl-*a*, Chl-*b*, and carotenoid concentrations by 37.74%, 84.85%, and 30.02%, respectively, in Pb-polluted soil (**Table 1**). Biochar alone increased Chl-*a*, Chl-*b*, and carotenoid synthesis by 32.07%, 57.57%, and 14.86%, respectively, while MT alone increased Chl-*a*, Chl-*b*, and carotenoid synthesis by 32.07%, 57.57%, and 14.86%, respectively (**Table 1**). Leaf RWC was significantly decreased by 33.48% than control in Pb-contaminated soil. However, BC and MT increased the leaf RWC, and the co-application of BC and MT showed more promising results than their individual application. The co-application of BC and MT increased RWC by 22.41%, while the individual BC and MT applications increased RWC by 13.18% and 8.13%, respectively (**Table 1**).

**Table 1:** Effects of biochar and melatonin on photosynthetic pigments and leaf water contents of rice in lead-polluted soil.

Treatments	Chl-*a* (mg g^−1^ FW)	Chl-*b* (mg g^−1^ FW)	Cart. (mg g^−1^ FW)	RWC (%)
Control	0.83a ± 0.040	0.67a ± 0.016	5.05a ± 0.057	84.40a ± 1.76
Pb stress	0.53c ± 0.016	0.33d ± 0.094	3.43d ± 0082	63.23d ± 0.85
Pb stress+BC	0.70b ± 0.017	0.52b ± 0.028	3.94b ± 0.086	71.57c ± 0.98
Pb stress+MT	0.66b ± 0.020	0.43c ± 0.020	3.71c ± 0.065	68.37c ± 1.51
Pb stress+BC+MT	0.73b ± 0.014	0.61a ± 0.041	4.46a ± 0.043	77.40b ± 1.60

The data are mean (n = 3) with ± SD. Different letters with means indicate the significance at p ≤ 0.05 according to Tukey’s honestly significant difference (HSD) test.

Chl, chlorophyll; Cart, carotenoid; RWC, relative water content; BC, biochar; MT, melatonin.

### Oxidative markers and antioxidant activities

The results revealed that plants growing in Pb-polluted soil faced a serious increase in EL, MDA, and H_2_O_2_ production. Nevertheless, BC and MT profoundly decreased the EL, MDA, and H_2_O_2_ production. EL, MDA, and H_2_O_2_ were increased by 192%, 93%, and 173%, respectively, under Pb stress than control (**Table 2**). BC+MT appreciably reduced EL, MDA, and H_2_O_2_ by 124%, 67.39%, and 74%, respectively (**Table 2**). Further, BC alone decreased EL, MDA, and H_2_O_2_ production by 74.33%, 32.75%, and 45.94%, respectively, while MT alone decreased EL, MDA, and H_2_O_2_ synthesis by 63.92%, 40.24%, and 29.25%, respectively (**Table 2**). The results depicted that Pb toxicity decreased TSP and FAA, while the synthesis of proline was increased in Pb stress. Further, BC and MT increased TSP, FAA, and proline synthesis (**Table 2**). The combined application of BC and MT increased TSP, FAA, and proline synthesis by 47.09%, 17.45%, and 32%, respectively (**Table 2**). We also noted that BC alone increased TSP, FAA, and proline synthesis by 34.69%, 17.60%, and 17.71%, respectively, while MT alone increased TSP, FAA, and proline synthesis by 16.32%, 8.04%, and 0.37%, respectively (**Table 2**). Antioxidant activities were slightly increased in Pb-contaminated soil; however, BC, MT, and BC+MT enhanced the activities of all the antioxidants (**Table 2**). The co-application of BC and MT enhanced APX, CAT, POD, and SOD activities by 26.47%, 65.51%, 93.10%, and 65.53%, respectively. Moreover, the individual BC application increased APX, CAT, POD, and SOD activities by 15.03%, 44.20%, 65.51%, and 46.46%, respectively, while the individual MT application increased by 8%, 37.30%, 20.68%, and 24.92%, respectively (**Table 2**).

**Table 2:** Effects of biochar and melatonin on oxidative markers, osmolyte synthesis, and antioxidant activity of rice in lead-polluted soil.

Treatments	EL (%)	MDA (µ mol g^−1^ FW)	H_2_O_2_ (µ mol g^−1^ FW)	TSP (mg g^−1^ FW)	FAA (mg g^−1^ FW)	Proline (mg g^−1^ FW)	APX (U/mg protein)	CAT (U/mg protein)	POD (U/mg protein)	SOD (U/mg protein)
Control	15.40d ± 0.82	3.59d ± 0.09	1.89e ± 0.10	10.49a ± 0.38	8.45a ± 0.34	0.540c ± 0.014	5.20e ± 0.07	2.72d ± 0.097	0.21c ± 0.21	2.54e ± 0.07
Pb stress	45.03a ± 1.75	6.93a ± 0.10	5.17a ± 0.14	6.37d ± 0.11	6.59c ± 0.14	0.807b ± 0.065	6.12d ± 0.10	3.19c ± 0.11	0.29bc ± 0.30	3.25d ± 0.11
Pb stress+BC	25.83b ± 0.48	5.22b ± 0.19	3.47c ± 0.06	8.58b ± 0.14	7.75ab ± 0.22	0.950b ± 0.049	7.04b ± 0.13	4.60b ± 0.19	0.48a ± 0.48	4.76b ± 0.06
Pb stress+MT	27.47b ± 0.82	4.95b ± 0.09	4.00b ± 0.18	7.41c ± 0.12	7.12bc ± 0.10	0.810b ± 0.021	6.61c ± 0.08	4.38b ± 0.11	0.35b ± 0.35	4.06c ± 0.11
Pb stress+BC+MT	20.07c ± 1.08	4.14c ± 0.14	2.97d ± 0.12	9.37b ± 0.37	7.74ab ± 0.23	1.127a ± 0.062	7.74a ± 0.14	5.28a ± 0.13	0.56a ± 0.56	5.38a ± 0.09

The data are mean (n = 3) with ± SD. Different letters with means indicate the significance at p ≤ 0.05 according to Tukey’s honestly significant difference (HSD) test.

EL, electrolyte leakage; MDA, malondialdehyde; H_2_O_2_, hydrogen peroxide; TSP, total soluble protein; FAA, free amino acids; APX, ascorbate peroxidase; CAT, catalase; POD, peroxidase; SOD, superoxide dismutase; BC, biochar; MT, melatonin.

### Pb and nutrient concentrations in plant parts

**Figure 2:**
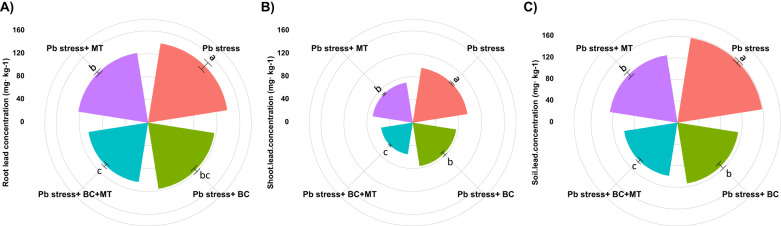
Effects of biochar and melatonin on root **(A)**, shoot **(B)**, and soil Pb **(C)** concentration of rice in Pb-polluted soil. The data are mean (n = 3) with ± SD, and different letters with means indicate the significance at p ≤ 0.05.

The presented results indicated that the Pb concentration significantly increased in rice roots and shoots growing in Pb-polluted soil (**Figure 2**). The maximum Pb concentration in the roots (139.67 mg kg^−1^) and shoots (96.37 mg kg^−1^) was detected in Pb-contaminated soil. BC+MT reduced Pb accretion in plant tissues, and the lowest root (104.80 mg kg^−1^) and shoot (55.77 mg kg^−1^) Pb were detected with BC+MT. Thereafter, BC showed better results, and it had the lowest root (116.70 mg kg^−1^) and shoot (76.40 mg kg^−1^) Pb concentrations, while MT alone had higher root (122.80 mg kg^−1^) and shoot (70.60 mg kg^−1^) Pb concentrations. We observed that Pb toxicity seriously decreased the tissue nutrient concentrations. The lowest root (9.67, 6.48, and 15.40 mg kg^−1^) and shoot (11.73, 9.81, and 18.54 mg kg^−1^) N, P, and K concentrations were found under Pb stress (**Table 3**). Biochar and MT increased the uptake of nutrients and resulted in better concentrations of these nutrients in roots and shoots. The co-application of BC and MT substantially enhanced root N, P, and K concentrations by 67.11%, 98.61%, and 100%. Further, BC enhanced N, P, and K concentrations by 53.46%, 68.05%, and 71.62%, respectively, while MT alone increased the root N, P, and K concentrations by 29.16%, 46.14%, and 48.63%, respectively (**Table 3**). The results also revealed that BC+MT enhanced shoot N, P, and K concentrations by 81.50%, 96.63%, and 108.78%, respectively. BC alone enhanced shoot N, P, and K concentrations by 62.23%, 59.73%, and 83.44%, and MT alone enhanced shoot N, P, and K concentrations by 53.70%, 31.80%, and 58.41%, respectively (**Table 3**). Pb toxicity also decreased the root and shoot Ca and Mg concentrations. The presented results indicated that the lowest root (41.40 and 33.93 mg kg^−1^) and shoot (56.40 and 44.43 mg kg^−1^) Ca and Mg contents were observed in Pb-contaminated soil, and the maximum root (70.03 and 62.47 mg kg^−1^) and shoot Ca (89.31 and 73.13 mg kg^−1^) concentrations in Pb-polluted soil were detected with the co-application of BC and MT and thereafter with the BC alone and MT alone applications (**Table 2**).

**Table 3:** Effects of biochar and melatonin on growth and yield traits of rice in lead-polluted soil.

Treatments	RL (cm)	RFW (g)	RDW (g)	PH (cm)	TPP	HKW	GY/pot (g)	BY/yield (g)	HI (%)
Control	63.43a ± 1.30	16.50a ± 0.90	7.81a ± 0.13	101a ± 2.45	10.66a ± 0.49	3.95a ± 0.07	63.45a ± 1.82	203.74a ± 3.48	31.15a ± 0.92
Pb stress	49.45c ± 0.80	7.74e ± 0.45	4.96e ± 0.07	79c ± 1.23	7.67c ± 0.47	2.01e ± 0.03	36.38e ± 0.098	145.37c ± 2.86	25.04c ± 0.97
Pb stress+BC	51.00bc ± 2.16	12.00c ± 0.36	6.41c ± 0.12	90b ± 1.29	9.33abc ± 0.95	3.05c ± 0.05	47.71c ± 1.62	177.33b ± 4.11	26.90bc ± 0.35
Pb stress+MT	46.33bc ± 1.25	9.78d ± 0.25	5.68d ± 0.17	84c ± 1.26	8.66bc ± 0.51	2.68d ± 0.09	42.40d ± 0.82	157.67b ± 6.24	26.91bc ± 0.55
Pb stress+BC+MT	55.00b ± 3.27	14.14b ± 0.34	7.22b ± 0.19	96b ± 1.89	9.67ab ± 0.46	3.67b ± 0.11	56.17b ± 1.70	195.78a ± 2.88	28.71ab ± 1.25

The data are mean (n = 3) with ± SD. Different letters with means indicate the significance at p ≤ 0.05 according to Tukey’s honestly significant difference (HSD) test.

RL, root length; RFW, root fresh weight; RDW, root dry weight; PH, plant height; TPP, tillers/plant; HKW, 100-kernel weight; GY, grain yield; BY, biological yield; HI, harvest index; BC, biochar; MT, melatonin.

### Translocation factor and biological accumulation coefficient of Pb

**Figure 3:**
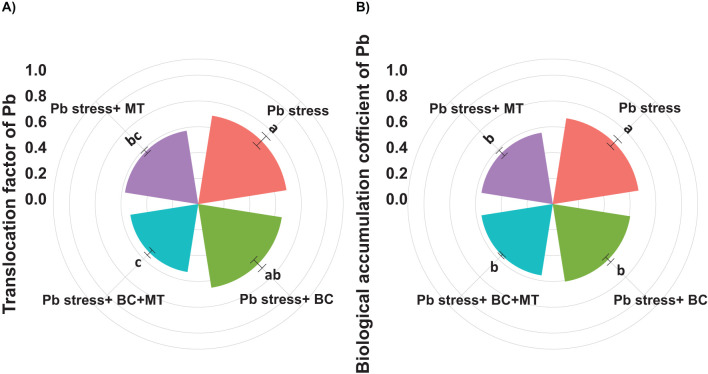
Effects of biochar and melatonin on translocation factor **(A)** and biological accumulation coefficient of Pb **(B)**. The data are mean (n = 3) with ± SD, and different letters with means indicate the significance at p ≤ 0.05.

The presented results revealed that BC and MT applications significantly decreased the TF and BAC of Pb (**Figure 3**). The maximum TF (0.693) and BAC (0.673) were observed in Pb-contaminated soil, followed by the MT alone (0.575 and 0.560) and BC alone (0.656 and 0.608) applications, and the lowest TA (0.533) and BAC (0.560) were observed with the combined BC and MT application (**Figure 3**).

### Soil properties

**Figure 4:**
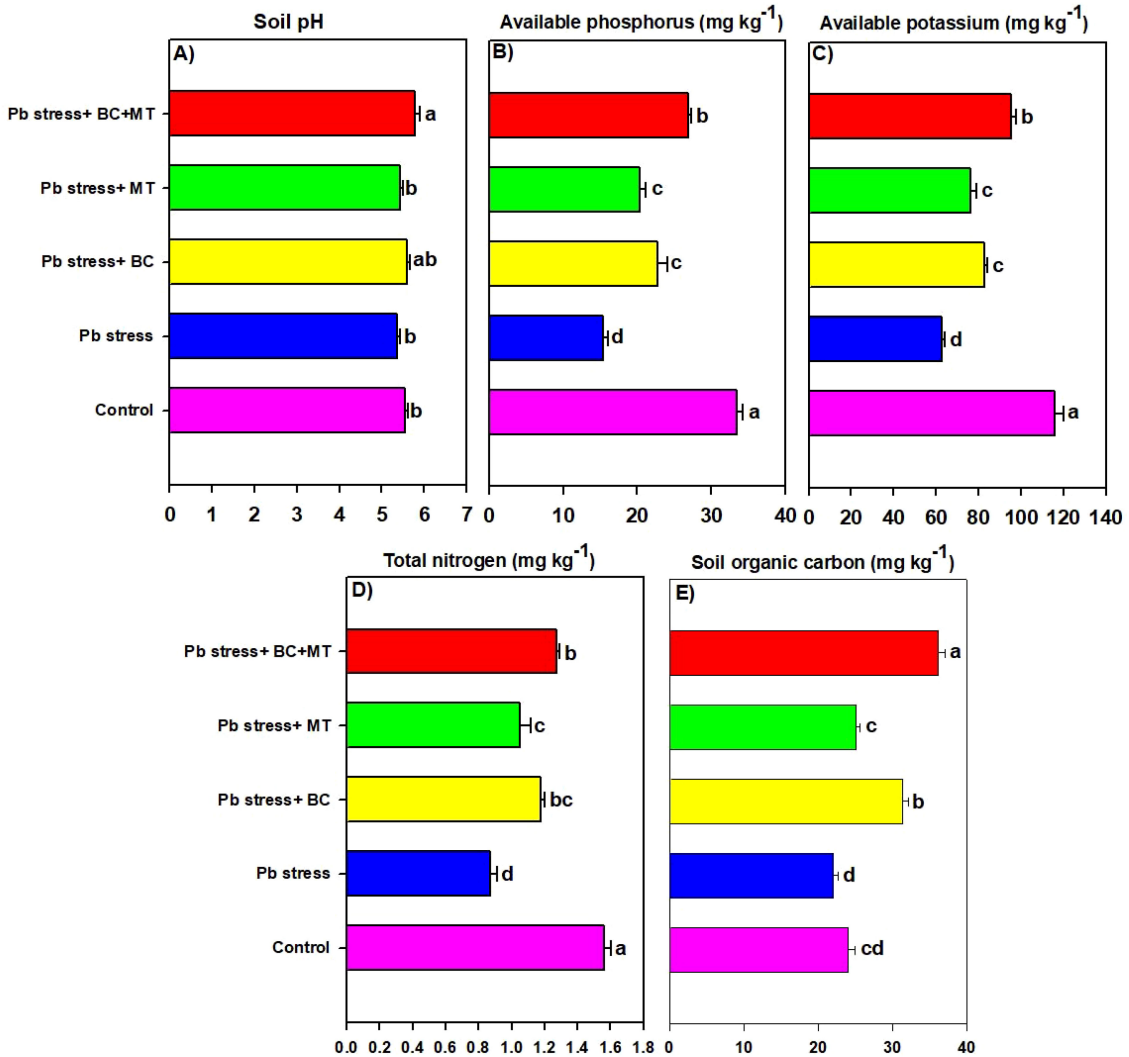
Effects of biochar and melatonin on soil pH **(A)**, AP **(B)**, AK **(C)**, TN **(D)**, and SOC **(E)**. The data are mean (n = 3) with ± SD, and different letters with means indicate the significance at p ≤ 0.05. AP, available phosphorus; AK, available potassium; TN, total nitrogen; SOC, soil organic carbon.

**Figure 5:**
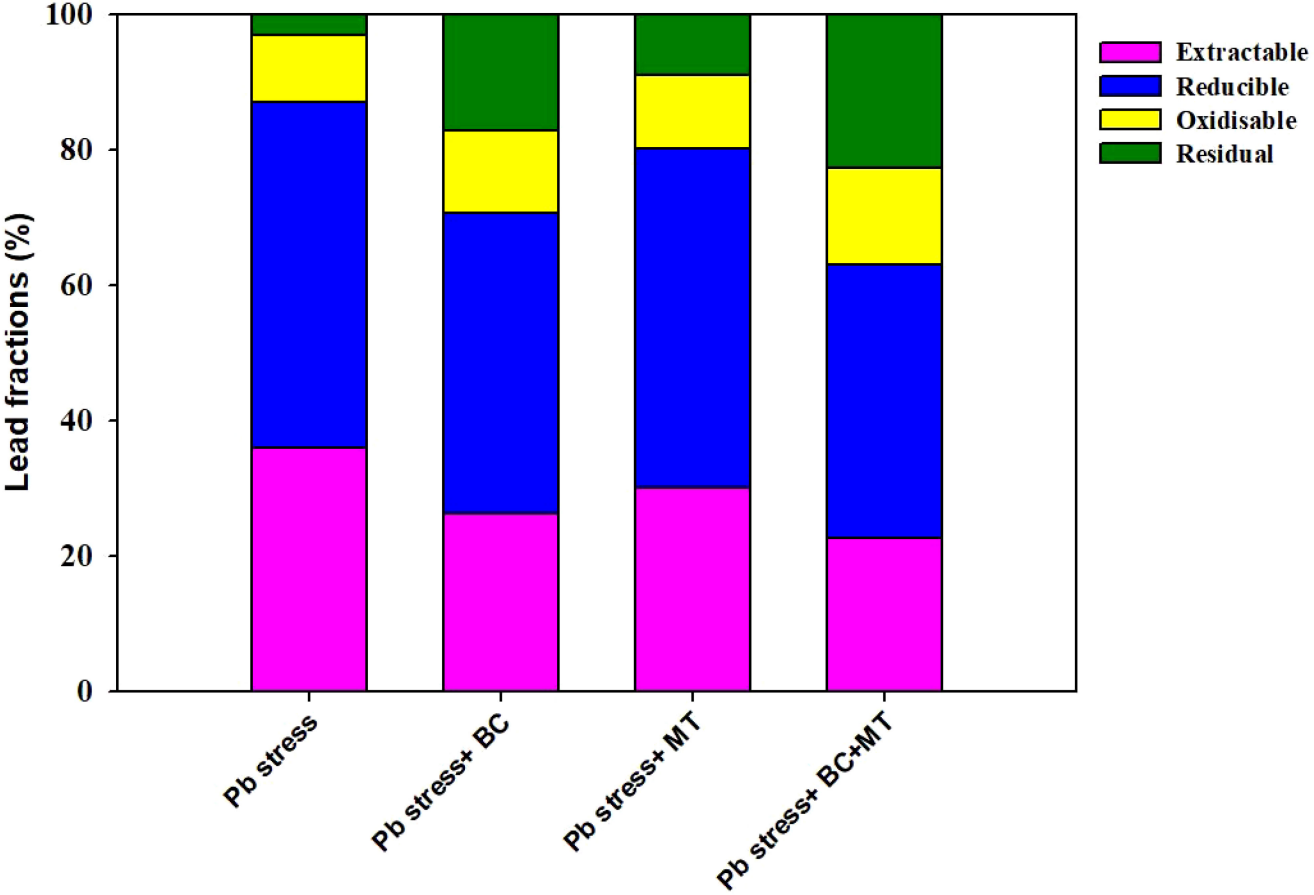
Effects of biochar and melatonin application on different fractions of Pb. The data are mean of three replications.

The maximum soil Pb concentration (158.50 mg kg^−1^) was found in Pb-contaminated soil, and the lowest soil Pb (99.67 mg kg^−1^) was observed with the co-application of BC and MT, followed by BC alone (113.63 mg kg^−1^) and MT alone (126.38 mg kg^−1^) (**Figure 2**). Different treatments induced a slight change in soil pH; however, BC and MT applications more effectively increased soil pH (**Figure 4**). N, P, and K availability was significantly decreased by 116.04, 84.88, and 78.69%, respectively, than the control. Nevertheless, the combined BC and MT application enhanced N, P, and K availability by 74.25%, 52.07%, and 45.81%, respectively, in Pb-polluted soil (**Figure 4**). Furthermore, BC alone enhanced soil N, P, and K availability by 35.16%, 47.28, and 31.72%, respectively, while MT alone enhanced soil, P, and K availability by 20.27%, 31.30%, and 21.55%, respectively (**Figure 4**). Soil organic concentration was significantly increased with BC application. The maximum SOC concentration (36.14 mg kg^−1^) in Pb-contaminated soil was obtained with the combined BC and MT application, followed by the BC alone (31.38 mg kg^−1^) and MT alone (25.11 mg kg^−1^) applications (**Figure 4**). BC+MT showed a significant impact on the Pb fractions (**Figure 5**). The results indicated that the BC+MT application significantly increased the residual and oxidizable forms of Pb, while BC+MT decreased the extractable and reducible forms of Pb (**Figure 5**). The maximum reducible and extractable fractions of Pb were observed in Pb-contaminated soil, whereas the lowest fractions were detected in soil that received BC+MT (**Figure 5**).

### Growth and yield traits

Root length (RL), root fresh weight (RFW), and root dry weight (RDW) were decreased by 28.27%, 113.17%, and 57.45%, respectively, under Pb stress (**Table 3**). Biochar, MT, and their combination significantly increased the RL, RFW, and RDW of rice plants (**Table 3**). The co-application of BC and MT increased RL, RFW, and RDW by 11.22%, 82.68%, and 45.56%, respectively; BC alone enhanced RL, RFW, and RDW by 3.12%, 55.03%, and 29.23%, respectively; MT alone enhanced RL, RFW, and RDW by 6.73%, 26.35%, and 14.51%, respectively (**Table 3**). The results also indicated that shorter plants (79 cm) with fewer tillers/plant (TPP) (7.67) and less 100-kernel weight (HKW) (2.01 g) were observed in Pb-polluted soil without any amendment, and taller plants (96 cm) with higher TPP (9.67) and HKW (3.67 g) were observed in Pb-contaminated soil with the co-application of BC and MT, followed by the BC application and then the MT alone application (**Table 3**). Pb toxicity also caused a reduction of 74.40%, 40.15%, and 24.40%, respectively, in grain yield (GY), biological yield (BY), and harvest index (HI) of rice crops (**Table 3**). Biochar, MT, and their combination appreciably increased the GY, BY, and HI of rice crops. The results indicated an increase of 54.39%, 33.30%, and 14.65% in GY, BY, and HI, respectively, with the combined BC and MT application in Pb-contaminated soil (**Table 3**). Moreover, BC alone enhanced GY, BY, and HI by 30.67%, 21.98%, and 4.16%, respectively, while MT alone enhanced GY, BY, and HI by 15.27%, 8.46%, and 0.73%, respectively (**Table 3**).

### Concentrations of Pb and iron in root surface iron plaques and relative expression of antioxidant defense and metal transporter genes

**Figure 6:**
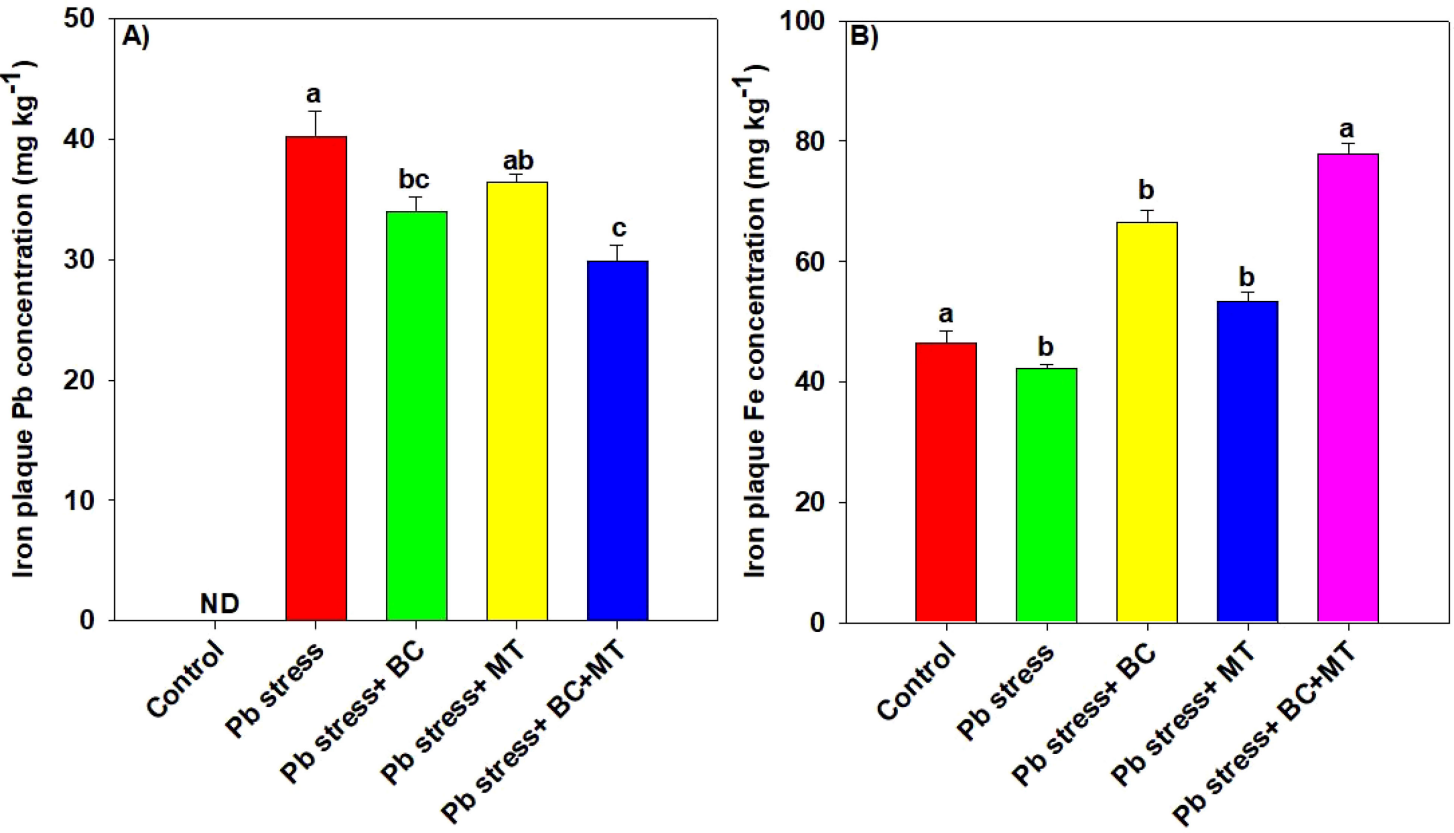
Effects of biochar and melatonin on concentrations of lead **(A)** and iron **(B)** in ACA extract of rice roots. The data are mean (n = 3) with ± SD, and different letters with means indicate the significance at p ≤ 0.05. ACA, ascorbic citrate acetic.

The results regarding the concentrations of Pb and Fe in the ACA extracts of the Fe plaque are given in **Figure 6**. The results revealed that the concentration of Pb significantly increased Pb in roots grown in Pb-polluted soil, while the application of both MT and BC significantly decreased the Pb concentration (50.14%) in ACA extracts of the Fe plaque of rice roots, while BC alone and MT alone decreased the Pb concentration by 18.69% and 10.60%, respectively (**Figure 6**). Notably, Fe concentration was significantly increased with BC and MT; however, a more significant increase (67.70%) in Fe concentration in ACA extracts of Fe plaque was observed with the co-application of BC and MT, followed by BC alone (57.21%) and MT (26.06%) (**Figure 6**).

**Figure 7:**
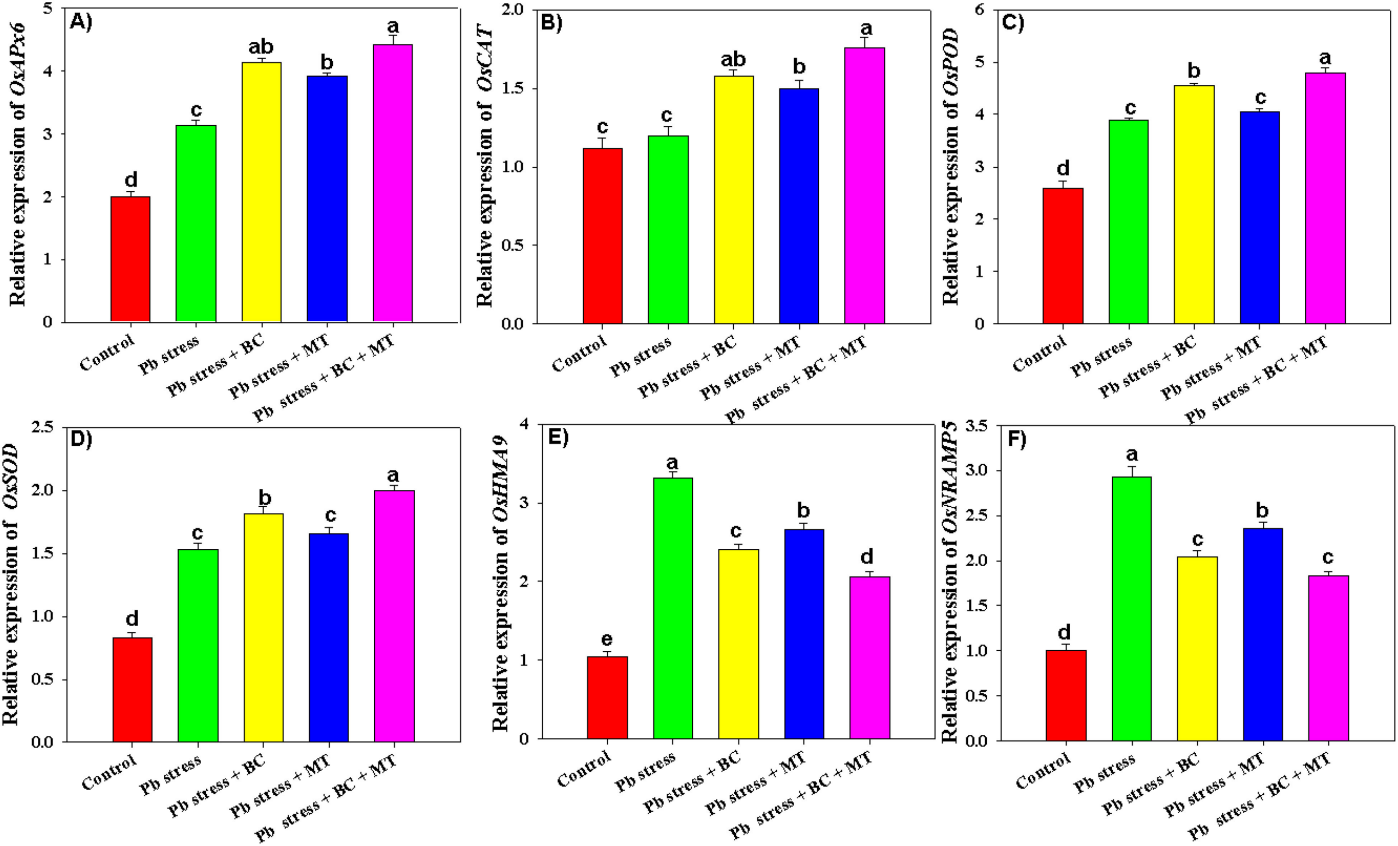
Effects of biochar and melatonin on expression level of antioxidant genes **(A–D)** and metal transporter genes **(E, F)** of rice grown under Pb stress. The data are mean (n = 3) with ± SD, and different letters with means indicate the significance at p ≤ 0.05.

The results indicated that BC, MT, and their combined application significantly impacted the expression of antioxidant defense genes (*OsAPX*, *OsCAT*, *OsPOX*, and *OsSOD*) and metal transporter genes (*OsHMA9* and *OsNRAMP5*). Pb toxicity decreased the expression of antioxidant defense genes; however, BC+MT significantly enhanced the expression of all the antioxidant genes (**Figure 7**). Co-applying BC+MT increased *OsAPX*, *OsCAT*, *OsPOX*, and *OsSOD* expression by 121%, 57.14%, 85.32%, and 140%, respectively, as compared to control (**Figure 7**). Moreover, BC alone increased *OsAPX*, *OsCAT*, *OsPOX*, and *OsSOD* expression by 106.50%, 41.07%, 49.80%, and 123.75%, respectively, while MT alone increased *OsAPX*, *OsCAT*, *OsPOX*, and *OsSOD* expression by 96%, 33.92%, 55.98%, and 100%, respectively, compared to control (**Figure 7**). The application of BC and MT also decreased the expression of the metal transporter genes, and more promising results were seen with the BC+MT application (**Figure 7**). Co-applying BC+MT decreased the expression of *OsHMA9* and *OsNRAMP5* by 96.19% and 83%, respectively, under Pb stress compared to control (**Figure 7**). Moreover, BC alone decreased *OsHMA9* and *OsNRAMP5* expression by 27.19% and 43.62%, respectively, while MT alone decreased *OsHMA9* and *OsNRAMP5* expression by 24.43% and 24.15%, respectively, than the control (**Figure 7**).

### Principal component and correlation analyses

**Figure 8:**
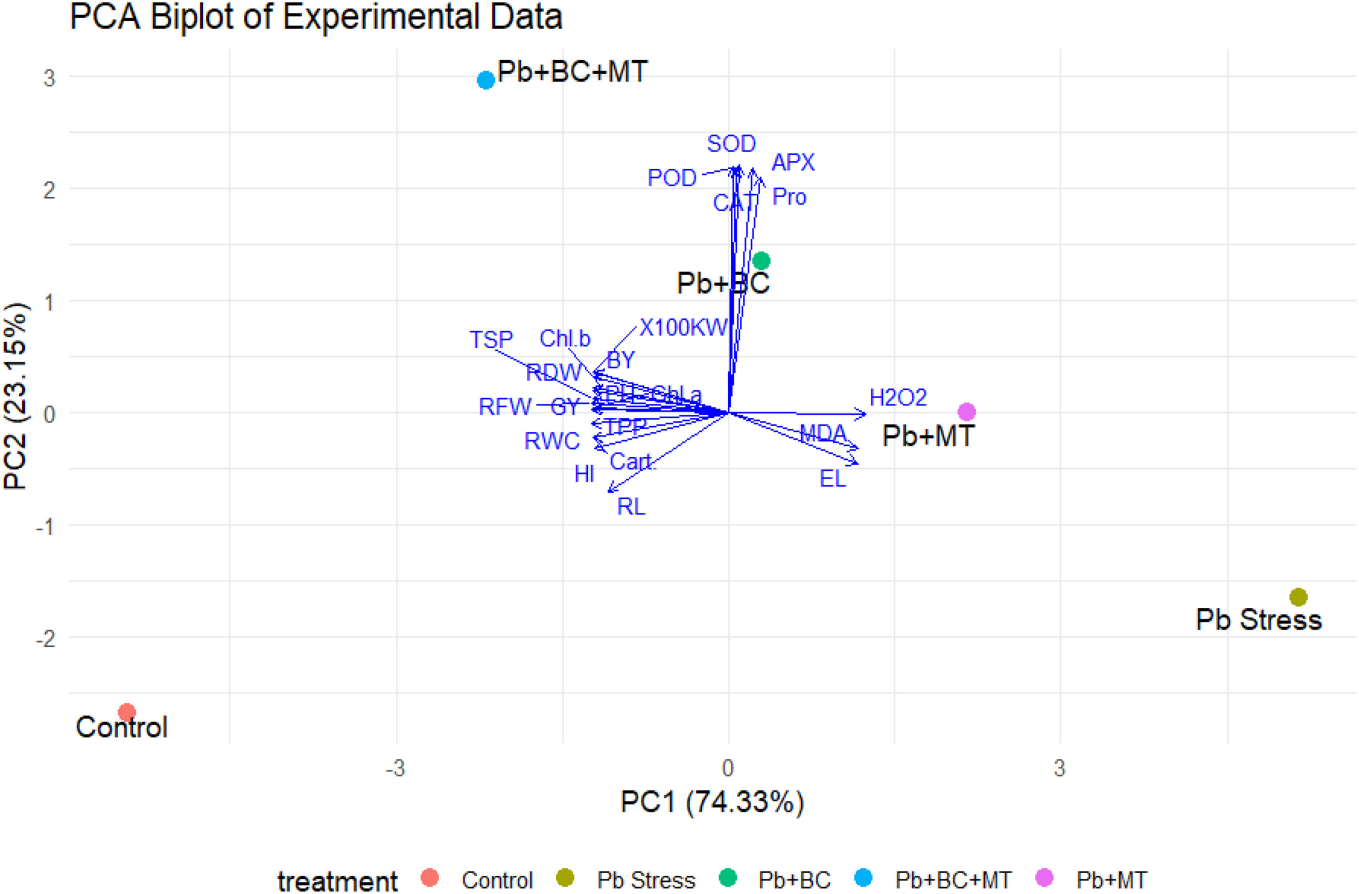
Principal component analysis indicating the impacts of different treatments on collected traits.

The PCA biplot explains 97.48% of the total variance, with PC1 accounting for 74.33% and PC2 accounting for 23.15%. The treatments exhibited distinct separation, with the control group clustering on the negative side of PC1, which correlated with growth parameters such as RL, BY, and HI. In contrast, Pb stress grouped on the positive side of PC1, which is closely associated with oxidative stress markers like MDA, EL, and H_2_O_2_. The Pb+BC and Pb+MT treatments showed moderate separation, with Pb+BC associated with antioxidant enzyme activity (APX, POD, and SOD) and Pb+MT linked to higher H_2_O_2_ levels. The combined treatment of Pb+BC+MT clustered centrally, indicating strong associations with antioxidant enzymes and proline, suggesting enhanced stress mitigation. Vector analysis revealed an inverse relationship between growth parameters and stress indicators, underscoring the effectiveness of BT+MT in alleviating Pb-induced stress and enhancing plant growth and physiological responses (**Figure 8**).

**Figure 9:**
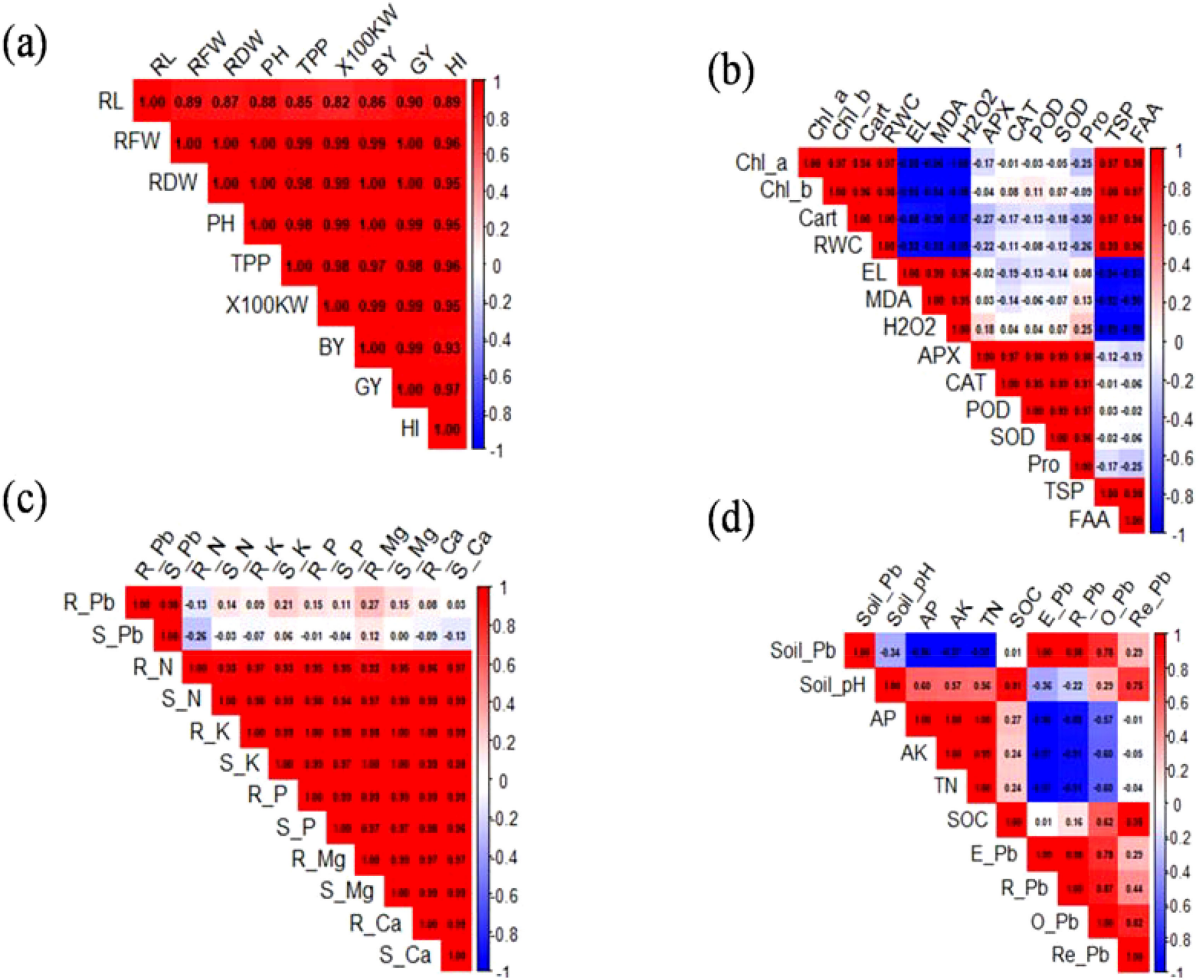
Pearson’s correlation analysis of growth **(a)**, physiological parameters **(b)**, root and shoot Pb and nutrient concentration **(c)**, and soil properties **(d)**. RL, root length; RFW, root fresh weight; RDW, root dry weight; PH, plant height; TPP, tillers/plant; KW, kernel weight; BY, biological yield; GY, grain yield; HI, harvest index; Chl, chlorophyll; Cart, carotenoids; RWC, relative water content; H_2_O_2_, hydrogen peroxide; MDA, malondialdehyde; APX, ascorbate peroxidase; CAT, catalase; POD, peroxidase; SOD, superoxide dismutase; pro, proline; TSP, total soluble proteins; N, nitrogen; P, phosphorous; K, potassium; Mg, magnesium; Ca, calcium; AP, available phosphorus; AK, available potassium; TN, total nitrogen; E-Pb, extractable Pb; R-Pb, reducible Pb; O-Pb, oxidizable Pb; Re-Pb, residual Pb.

The correlation matrix for growth parameters is depicted in **Figure 9a**. A strong and significant positive correlation was observed among all growth traits, including RL, RFW, RDW, PH, TPP, HKW, BY, GY, and HI. The r values ranged from 0.86 to 1.00, indicating a very close association among these parameters. Notably, RDW, BY, and GY showed particularly strong correlations (r > 0.97) with HI, highlighting their importance as determinants of harvest index performance. The correlation among physiological traits is depicted in **Figure 9b**. Chl-*a*, Chl-*b*, and carotenoids were strongly positively correlated (r = 0.93–1.00). The RWC was negatively correlated with MDA (r = −0.50), EL (r = −0.58), and H_2_O_2_ (r = −0.52), indicating that increased oxidative stress results in reduced water retention. Antioxidant enzymes, such as APX, CAT, POD, and SOD, exhibited weak to moderate correlations, with oxidative stress markers having r values ranging from −0.11 to 0.37. Proline (Pro) showed a weak negative association with EL and oxidative stress markers (r = −0.17 to −0.25).

R-Pb and S-Pb were strongly correlated with N, K P, Mg, and Ca levels in plant tissues. Root and shoot Pb concentrations were positively correlated with R-K, R-P, S-K, and S-P (r > 0.90), indicating a coordinated uptake pattern. In contrast, R-Pb exhibited a weak negative correlation with N uptake (r = −0.26; **Figure 9c**). These patterns suggest that Pb accumulation may disrupt specific nutrient dynamics, particularly N. The relationships among soil properties are illustrated in **Figure 9d**. The soil Pb concentration was negatively correlated with the soil pH (r = −0.54), indicating that acidic conditions increase Pb bioavailability. SOC and TN contents were weakly negatively associated with the Pb fraction (r = −0.31 to −0.46). Exchangeable and reducible Pb fractions were strongly correlated (r = 0.78) and positively associated with total soil Pb content (r = 0.73). Interestingly, AP and AK were moderately positively correlated with the soil Pb concentration (r = 0.57).

## Discussion

Pb is a non-essential metal, yet its accumulation is increasing in soil because of human actions (Alengebawy et al., 2021; Fatemi et al., 2021). In this study, Pb toxicity (300 mg kg^−1^) seriously inhibited the rice growth (**Table 3**). This could be attributed to impaired chlorophyll synthesis (**Table 1**), reduced leaf water contents, inhibited root growth (**Table 3**), and compromised cell division (Fatemi et al., 2021). Pb stress decreased root growth, which was a reason for the reduction in growth. Roots are directly exposed to Pb, which disrupts the physiological functioning of roots and the mobilization of minerals and water, thereby leading to poor growth (Vasilachi et al., 2023). Poor root growth also disturbed the nutrient uptake, resulting in a substantial reduction in nutrient accumulation in plant tissues (**Table 4**). These findings align with previous studies where authors documented that Pb toxicity reduces nutrient uptake, which adversely impacts ATP productivity, enzyme activity, and biochemical function, thereby leading to poor plant growth (Aslam et al., 2021; Vasilachi et al., 2023).

**Table 4:** Effects of biochar and melatonin on nutrient concentration in roots and shoots of rice in lead-polluted soil.

Treatments	Root-N	Shoot-N	Root-P	Shoot-P	Root-K	Shoot-K	Root-Ca	Shoot-Ca	Root-Mg	Shoot-Mg
mg kg^−1^ DW										
Control	12.74b ± 1.21	14.52c ± 0.71	7.86cd ± 0.32	11.70cd ± 0.29	19.74d ± 0.42	22.27d ± 0.86	49.45c ± 2.45	68.50c ± 2.12	36.90d ± 2.05	51.67d ± 1.94
Pb stress	9.67c ± 0.20	11.73d ± 0.48	6.48d ± 0.28	9.81d ± 0.33	15.40e ± 0.83	18.54e ± 0.46	41.10d ± 1.95	56.40d ± 3.48	33.93d ± 0.66	44.43e ± 1.59
Pb stress+BC	14.84ab ± 0.48	19.03b ± 0.34	10.89ab ± 0.45	15.67b ± 0.41	26.43b ± 0.65	34.01b ± 0.50	60.20b ± 1.75	81.70b ± 2.17	55.47b ± 0.78	66.87b ± 1.32
Pb stress+MT	12.49b ± 0.23	18.03b ± 0.39	9.47bc ± 0.58	12.93c ± 0.42	22.89c ± 0.45	29.37c ± 0.69	56.37b ± 2.24	75.31bc ± 2.56	47.75c ± 2.40	60.60c ± 0.82
Pb stress+BC+MT	16.16a ± 1.02	21.29a ± 0.92	12.87a ± 1.25	19.29a ± 1.06	30.44a ± 0.68	38.71a ± 1.64	70.03a ± 1.55	89.31a ± 1.63	62.47a ± 1.67	73.13a ± 1.79

The data are mean (n = 3) with ± SD. Different letters with means indicate the significance at p ≤ 0.05 according to Tukey’s honestly significant difference (HSD) test.

N, nitrogen; P, phosphorus; K, potassium; Ca, calcium; Mg, magnesium; BC, biochar; MT, melatonin.

The co-application of BC and MT enhanced rice growth and productivity (**Table 3**). Biochar enhanced chlorophyll synthesis and maintained leaf water content and membrane stability by decreasing ROS (**Table 2**) through a substantial increase in antioxidant activity and osmolyte synthesis. In addition, BC and MT also increased nutrient availability, and SOC consequently decreased the availability of Pb, leading to improved growth and yield (Li et al., 2023). The biochar used in the present study contained significant amounts of micronutrients and macronutrients, which hold significant value for enhancing crop productivity and soil fertility (Ahmad et al., 2023). Melatonin has emerged as an excellent stress protectant. The foliar-applied MT considerably enhanced rice productivity, which could be ascribed to enhanced chlorophyll synthesis, better ROS scavenging, and nutrient availability (Sun et al., 2023; Altaf et al., 2024). The improvements in rice yield were also credited to a substantial decrease in Pb accumulation and an increase in proline, TSP, and FAA synthesis following MT application (Song et al., 2024). Melatonin application also enhanced root growth and improved nutrient absorption, which supported rice seedlings, thereby ensuring better plant growth (Buttar et al., 2020; Yang et al., 2023b). Melatonin application also facilitated nutrient uptake and reduced soil-available Pb and its accumulation in plant tissues, thereby ensuring better growth (Seth et al., 2023).

Pb stress seriously inhibited chlorophyll synthesis (**Table 1**), which was associated with reduced stomatal conductance and increased oxidative damage (Kaya et al., 2019). Pb toxicity may also destroy the chloroplast infrastructure and increase chlorophyll-degrading enzyme activity, thereby leading to a reduction in chlorophyll synthesis (Ghori et al., 2019). Pb stress also decreased the RWC (**Table 1**), which was associated with the increased accumulation of Pb in plant tissues, reducing water uptake from soil and resulting in water scarcity (Haider et al., 2021). Pb toxicity also reduces water absorption by lowering root hydraulic conductivity, which can decrease cell turgor and RWC. This finding aligns with previous studies where authors witnessed that Pb toxicity decreased water uptake by plants (Rasool et al., 2020). However, BC and MT significantly increased chlorophyll synthesis as compared to control. Therefore, BC and MT could reduce oxidative damage (**Table 3**) and shield the photosynthetic apparatus, thereby leading to increased chlorophyll synthesis. BC could also reduce the Pb uptake by plants, leading to enhanced chlorophyll synthesis and photosynthetic efficiency (Mahamood et al., 2023). Melatonin application also upregulated the expression of different genes (CB12 and CAB7), which enhanced chlorophyll synthesis under HM stress (Ahmad et al., 2023; Altaf et al., 2024). The better RWC with BC and MT was also associated with good root growth (**Table 1**), which increased the water uptake and resulted in better water contents in plant tissues.

In this study, Pb toxicity significantly increased the expression of oxidative stress markers, which reduced growth (Abu-Shahba et al., 2022; Rady et al., 2023). ROS induced MDA and EL; in contrast, BC and MT applications significantly decreased EL, H_2_O_2_, and MDA by increasing antioxidant activity and osmolyte synthesis and reducing Pb availability (Yang et al., 2023b). Biochar and MT also facilitated nutrient uptake (**Table 4**), which increased the functioning of antioxidant enzymes and, therefore, decreased EL, H_2_O_2_, and MDA production (Sun et al., 2022; Mahamood et al., 2023). Pb toxicity seriously decreased TSP and FAA contents, which was associated with N uptake (**Figure 3**) and its accumulation, which is essential for the synthesis of proteins and amino acids. However, proline synthesis was enhanced in Pb stress. Plants accumulate proline under HM stress, which improves the adjustment and protects the cellular membranes (El Rasafi et al., 2022).

Antioxidant activities increased under Pb stress; furthermore, BC and MT also boosted antioxidant activities. Plant defenses become more responsive when plants are grown in HM-polluted soil (Noor et al., 2022). The biochar and MT applications substantially enhanced antioxidant activity, which mitigated oxidative damages evidenced by lower EL, H_2_O_2_, and MDA production. MT application improved antioxidant activities, which facilitated redox homeostasis and mitigated the adverse impacts caused by HMs (Malik et al., 2022). We found that APX activity was significantly higher than POD activity. APX is more induced under oxidative stress compared to POD. This is linked to the fact that APX is part of a water–water cycle in chloroplasts and the cytosol, where rapid H_2_O_2_ removal is crucial to prevent cellular damage (Yoshimura and Ishikawa, 2024). Conversely, POD is more associated with secondary metabolic processes. The combined application of BC and MT decreased MDA and H_2_O_2_ synthesis, which may have led to a decrease in POD demand; thus, APX activity was higher than POD activity. Biochar application enhanced all the antioxidant activities, which exerted positive impacts on plant growth. This occurred because Pb toxicity enhanced oxidative damage, and in turn, BC increased antioxidant activity by increasing nutrient uptake, which mitigated Pb toxicity (Irshad et al., 2020a).

Pb accumulation increased in plant tissues growing in Pb-polluted soil (**Figure 2**). The maximum Pb concentration was detected in roots, and the lowest was recorded in above-ground parts. This was observed as a key mechanism employed by rice plants to mitigate Pb toxicity. The co-application of BC and MT significantly decreased Pb in shoots and grain than in roots. This reduction was linked with the “dilution effect”, where BC and MT enhanced rice growth and led to a dilution of Pb in plant parts. In addition to this, the increased Pb accretion in roots may also be associated with the Pb precipitation in inter-cellular spaces, its vacuolar and cortical cell sequestrations, and cortical cells (Murtaza et al., 2019). Biochar in combination with MT significantly decreased Pb accumulation in plant tissues. Biochar increases soil pH (**Figure 5**), which may increase the sorption of Pb, thereby reducing its accumulation in plant tissues (Fu et al., 2023). The biochar and MT applications also reduced the TF and BAC values, which aligns with earlier studies that support that BC decreases the uptake of Pb in plants (Chen et al., 2023). The presence of oxygen-containing functional groups in BC also immobilizes Pb, therefore decreasing Pb availability and subsequent accretion in plants (Zhang et al., 2020).

The biochar and MT applications significantly influenced the soil properties after harvesting rice crops (**Figure 5**). The results revealed that BC and BC+MT significantly increased the pH of Pb-contaminated soil; however, the MT alone had a non-significant impact on soil pH (**Figure 4**). Biochar had a pH of 9.72 and a porous structure (**Figure 1**), and it may contain hydroxides, carbonates, organic anions, and ash, which have limiting impacts, thereby leading to an increase in soil pH (Wang et al., 2019). In this study, BC also increased SOC, which was associated with the presence of carbon in BC and an increase in soil pH. The higher pH imposes a greater negative charge, which facilitates dissolved organic carbon (DOC) desorption, thereby increasing soil carbon contents (El-Naggar et al., 2020). Biochar and MT significantly increased N, P, and K uptake; however, more reliable results were seen with the BC+MT application. The increase in K and N availability was also correlated with a rise in the availability of these nutrients with the BC and MT applications. The increase in root growth following the BC and MT applications could also be a reason for increased nutrient availability in Pb-polluted soil. We also noted that BC enhanced the nutrient availability and decreased the Pb availability. Biochar improved soil conditioning and increased nutrient availability by slow release of nutrients (Yadav et al., 2023: Bekchanova et al., 2024). Furthermore, BC also caused the complexation and immobilization of Pb and decreased its availability and uptake by plants (Chen et al., 2022b; Ji et al., 2022). Therefore, BC used in the current study may cause the complexation and immobilization of Pb; thus, it increases nutrient uptake and availability while decreasing Pb availability and uptake. BC+MT decreased the reducible and extractable forms of Pb. Biochar has an excellent surface area that adsorbs Pb on its surface, and it also alters the soil pH, thereby reducing solubility and precipitating it as less soluble compounds (Yang et al., 2021). Biochar application increases soil pH, which decreases its solubility, thereby decreasing its availability and extractability in soil. Additionally, BC also promotes the precipitation of Pb as less mobile compounds, which decreases its extractability.

Iron plaques are formed around the outer cells of roots by the precipitation of iron oxide and inhibit the uptake of heavy metals, thereby reducing their toxicity to plants (Zheng et al., 2022; Zandi et al., 2023). In the present study, BC and MT, particularly their co-application, increased the formation of iron plaques on rice roots. Co-applying BC and MT substantially increased the ACA-extractable concentration of Fe and decreased the Pb concentration in rice roots (**Figure 6**). The combined application of BC and MT enhanced iron plaque accumulation, which plays a protective role on the root surface and reduced the absorption and transportation of Pb (Siddique et al., 2021). Additionally, Fe plaque co-precipitates and adsorbs heavy metals near roots, making it difficult for toxic metals to enter the inner layers of roots, which in turn protects plants from toxic metals (Zhang et al., 2021).

The results indicated that the expression of metal uptake genes (*OsHMA9* and *OsNRAMP5*) significantly increased under Pb toxicity (**Figure 5**). Both *OsHMA9* and *OsNRAMP5* play crucial roles in the detoxification of metals, and *OsHMA9* is mostly expressed in xylem and phloem tissues and plays a vital role in heavy metal uptake and transportation (Chang et al., 2022). *OsHMA9* plays a crucial role in the detoxification of toxic metals in unfavorable environments. This gene helps to sequester the toxic metals, including Pb in roots, therefore detoxifying and mitigating the adverse impacts of Pb in plants (Lee et al., 2007). *OsNRAMP5* is a major transporter of Pb, and its knockout in rice can decrease the Pb uptake by roots and its accumulation in root and aerial plant parts (Chang et al., 2022). Lead stress increased the expression of *OsHMA9*, while both BC and MT decreased *OsHMA9*. This may decrease the Cd uptake and accumulation in roots, thereby reducing its toxicity. Pb stress increased the *OsNRAMP5* expression, while BC and MT decreased its expression. This, in turn, decreased Pb uptake and its subsequent accumulation, which aligns with earlier studies by Chang et al. (2022). Pb toxicity increased the expression of antioxidant defense genes, and BC and MT applications further increased the expression of antioxidant defense genes (*OsAPX*, *OsCAT*, *OsPOX*, and *OsSOD*). These findings align with earlier findings, indicating that MT application increases gene expression to counter heavy metal toxicity (Jahan et al., 2020; Fu et al., 2022). The increase in gene expression after the MT+BC application modulates plant morpho-physiological and biochemical functions and decreases heavy metal uptake, thereby ensuring better plant growth under stress conditions (Shar et al., 2024).

## Conclusion

Lead stress decreased rice yield by disturbing plant functions, nutrient availability, and soil properties and increasing Pb accumulation in plant parts. The combination of BC and MT improved antioxidant activities, chlorophyll synthesis, osmolyte accumulation, and the expression of antioxidant genes and reduced oxidative stress markers, thereby leading to increased rice growth and yield. The combination of BC and MT also decreased soil Pb availability and Pb uptake and accumulation by decreasing the expression of metal transporter genes, leading to improved plant growth. This study revealed that combining BC and MT is a promising strategy to increase rice productivity in Pb-polluted soil. However, more transcriptome and metabolomics studies are needed to validate these results. Furthermore, field studies are also needed in contaminated areas to validate these results before they are recommended. The findings of the present study can also be applied in phyto-remediation areas because BC increases phyto-remediation potential, and BC application in tolerant plants can offer more promising results. In the future, authors should also perform a study to determine the impacts of BC alone and MT alone on rice performance in unpolluted soils.

## Data Availability

The original contributions presented in the study are included in the article/**Supplementary Material**. Further inquiries can be directed to the corresponding authors.
