# Supplementary material for: Integrative biochar and melatonin application mitigates lead toxicity in rice by modulating antioxidant activities and iron plaque formation and downregulating the expression of metal uptake genes
Source: Front Plant Sci. 2025 Jul 3;16:1609825. doi: 10.3389/fpls.2025.1609825 (PMC12267010; doi:10.3389/fpls.2025.1609825)
Supplement: Supplementary file 1 [file Table1.docx]

**Integrative Biochar and Melatonin Application Mitigates Lead Toxicity in Rice by Modulating Antioxidant Activities, Iron Plaque Formation and Down Regulating the Expression of Metal Uptake Genes**

Tahir Abbas Khan ^1^, Su Qitao ^2*^, Huang Guoqin ^1,3*^, Du Zhixuan ^2,3^, Mehmood Ali Noor ^1^, Tahani A.Y. Asseri ^4^, Muhammad Umair Hassan ^1^

^1^Research Center on Ecological Sciences, Jiangxi Agricultural University, Nanchang 330045, China

^2^School of Life Sciences, Key Laboratory of Jiangxi Province for Biological Invasion and Biosecurity, Jinggangshan University, Ji’an 343009, China

^3^Key Laboratory of Crop Physiology, Ecology and Genetic Breeding, Ministry of Education, Jiangxi Agricultural University, Nanchang 330045, China

^4^King Khalid University, College of Science, Department of Biology, Abha 61413, Saudi Arabia

*Corresponding authors: [suqitao@jgsu.edu.cn](mailto:suqitao@jgsu.edu.cn) and [hgqmail441@sohu.com](mailto:hgqmail441@sohu.com)

**Table S1: List of primers used for gene expression analysis**

| **Gene** | **Details of primers** |
| --- | --- |
| OsPOX-F | GAAGGGTTGATGTTGCTGCC |
| OsPOX-R | TCGGCGTTCTTTGATGTCCT |
| OsCAT-F | GCCACGAAGGACTTGACTGA |
| OsCAT-R | GAGATCCAGATGCCACGGAG |
| OsSOD-F | TACGGGTAGGGCACTGAACA |
| OsSOD-R | CTCCTTTCCGGCAGGATTGT |
| OsAPX-F | CCTTCACCTGCGGAACATCT |
| OsAPX-R | AGCACAGCATCAGTAGGCAG |
| OsHMA9-F | CAGTGAGCATCCTCTGGCAA |
| OsHMA9-R | GATGCTCCCGTCCTTTTGGA |
| OsNRAMP5-F | GAAGTGGCTTCGGAACCTGA |
| OsNRAMP5-R | GAAGCTCGTGCTCAGGAAGT |
| Actin-F | CATTGGTGCTGAGCGTTTCC |
| Actin-R | CCCGCAGCTTCCATTCCTAT |
